# Procalcitonin Identifies Cell Injury, Not Bacterial Infection, in Acute Liver Failure

**DOI:** 10.1371/journal.pone.0138566

**Published:** 2015-09-22

**Authors:** Jody A. Rule, Linda S. Hynan, Nahid Attar, Corron Sanders, William J. Korzun, William M. Lee, Acute Liver Failure Study Group

**Affiliations:** 1 Department of Internal Medicine, Division of Digestive Diseases, UT Southwestern Medical Center, Dallas, Texas, United States of America; 2 Department of Clinical Sciences and Department of Psychiatry, Division of Biostatistics, UT Southwestern Medical Center, Dallas, Texas, United States of America; 3 Department of Clinical Laboratory Sciences, School of Allied Health, Virginia Commonwealth University, Richmond, Virginia, United States of America; University Hospital Oldenburg, GERMANY

## Abstract

**Background:**

Because acute liver failure (ALF) patients share many clinical features with severe sepsis and septic shock, identifying bacterial infection clinically in ALF patients is challenging. Procalcitonin (PCT) has proven to be a useful marker in detecting bacterial infection. We sought to determine whether PCT discriminated between presence and absence of infection in patients with ALF.

**Method:**

Retrospective analysis of data and samples of 115 ALF patients from the United States Acute Liver Failure Study Group randomly selected from 1863 patients were classified for disease severity and ALF etiology. Twenty uninfected chronic liver disease (CLD) subjects served as controls.

**Results:**

Procalcitonin concentrations in most samples were elevated, with median values for all ALF groups near or above a 2.0 ng/mL cut-off that generally indicates severe sepsis. While PCT concentrations increased somewhat with apparent liver injury severity, there were no differences in PCT levels between the pre-defined severity groups–non-SIRS and SIRS groups with no documented infections and Severe Sepsis and Septic Shock groups with documented infections, (p = 0.169). PCT values from CLD patients differed from all ALF groups (median CLD PCT value 0.104 ng/mL, (p ≤0.001)). Subjects with acetaminophen (APAP) toxicity, many without evidence of infection, demonstrated median PCT >2.0 ng/mL, regardless of SIRS features, while some culture positive subjects had PCT values <2.0 ng/mL.

**Summary/Conclusions:**

While PCT appears to be a robust assay for detecting bacterial infection in the general population, there was poor discrimination between ALF patients with or without bacterial infection presumably because of the massive inflammation observed. Severe hepatocyte necrosis with inflammation results in elevated PCT levels, rendering this biomarker unreliable in the ALF setting.

## Introduction

Patients with acute liver failure (ALF) have an increased risk of infection approaching 80% thus predisposing to sepsis-related multi-organ failure [1–3]. Infections have been shown to worsen hepatic encephalopathy (HE) and are one of the most common causes of multi-organ dysfunction and death in ALF [3, 4].

Signs and symptoms of ALF closely mimic those of sepsis making it difficult to accurately identify infection in this setting. The diagnosis of infection or sepsis (usually based upon a positive culture) is often delayed or not detected, even when presumptive evidence is very strong. Identification of a bacterial infection by culture requires 48–72 hours to complete and the efficacy of prophylactic antibiotics to contain an established infection remains difficult to prove. A more rapid method to identify infection is a worthwhile goal.

Procalcitonin (PCT) is a biomarker for detection of bacterial infection that has shown early promise. PCT consists of 116 amino acids and has a MW of 14.5 kDa. A precursor of calcitonin, the primary source of PCT is the parafollicular C cells of the thyroid gland. Calcitonin has a hypocalcemic and hypophosphatemic effect that inhibits bone resorption and stimulates excretion of phosphorus, calcium, and sodium by the kidney and is used as a biomarker of medullary thyroid cancer but its relation to systemic inflammation remains unclear [5–8].

In studies of patients with sepsis, pneumonia, and other types of infections, PCT has been shown to be a reliable and rapidly available indicator of bacterial infection, useful in guiding antimicrobial therapy. PCT has been examined in patients who have had a liver transplant and in patients hospitalized with chronic liver disease (cirrhosis; CLD), but there are few studies of patients with ALF. [9–14].

In this study, we sought to determine whether PCT would aid in the identification of infection in ALF patients, since distinguishing sepsis from ALF without infection has prognostic and therapeutic implications. We utilized the extensive registry and serum bank of the U. S. Acute Liver Failure Study Group (USALFSG) as well as 2 local repositories for CLD control sera, to address this question.

## Materials and Methods

### Study Population

This study included patients prospectively enrolled in the US Acute Liver Failure Study (established in January 1998). Additionally, data and sera from two other repositories related to CLD were accessed to provide control groups. A total of 1863 adult ALF patients were enrolled from 23 tertiary care centers within the US between Jan. 1998 and Oct. 2010. Inclusion criteria defining ALF were the presence of coagulopathy (PT > 15 seconds or INR ≥1.5 IU) and any grade of hepatic encephalopathy (HE) that had occurred within 26 weeks of the first onset of symptoms. Enrolled patients may not have any CLD or cirrhosis, with the exception of certain patients with chronic hepatitis B or Wilson’s disease [15]. Written informed consent was obtained for each subject from the next of kin because of altered mental status due to HE. All centers were in compliance with their local institutional review board requirements. After informed consent was obtained, serum samples and clinical data were collected daily for up to 7 consecutive days. Sample collection ended when one of the following events occurred: the subject was discharged, transplanted, died, or completed the 7th study day. Serum samples were stored frozen at -70° until testing.

The Chronic Liver Disease (CLDD; PI–William M. Lee, MD) and the Nucleic Acid, Serum, and Tissue Repository for the Study of Liver Diseases Database and sample repositories (NSTLD; PI–Marlyn Mayo, MD) are principal investigator-initiated studies at UT Southwestern Medical Center (UTSW) in Dallas, TX. After written informed consent was obtained, sera were obtained and frozen at -70° until testing.

This retrospective analysis received Institutional Review Board (IRB) approval from the University of Texas Southwestern Medical Center (IRB#STU092010-126) and Virginia Commonwealth University (IRB#HM13517).

All study subjects were categorized into infection groups using modified definitions from the 2001 SCCM/ESICM/ACCP/ATS/SIS International Sepsis Definitions Conference definitions of systemic inflammatory response syndrome (SIRS), severe sepsis, and septic shock [9, 10, 16] based upon available data from the USALFSG database. Subjects with *SIRS* had to have two or more of the following signs or symptoms: body temperature >38°C or <36°C, heart rate >90 bpm, respiration rate >20 breaths/min, PCO_2_ level <32 mm Hg, WBC >12,000 or <4,000, or bands (immature granulocytic white blood cells) >10%. *Severe sepsis* was defined as organ failure in the presence of sepsis (SIRS plus a documented infection defined as a positive culture (blood, trachea, wound, catheter or urine) up to 2 days prior to the blood sample). *Septic shock* was defined as the presence of severe persistent hypotension (mean arterial pressure <60 mmHg) despite adequate volume resuscitation and pressor therapy in the presence of sepsis [16]. Non-infected/non-SIRS (non-SIRS) samples were selected from patients who met no more than one of the SIRS criteria.

For the 1,863 patients enrolled in the ALF study, 12,492 days of demographic and bio-data were sorted into 1 of the 4 groups: non-SIRS (n = 628), SIRS (n = 1407), severe sepsis (n = 387), and septic shock (n = 31). Because the USALFSG is an observational study not specifically designed to study SIRS or infections, some data parameters noted in the 4 different SIRS categories were either not captured by the ALF CRF or were not routinely collected on all patient days (examples: cell differential, urine output, tachypnea, and cardiac index). Thus, each patient day was categorized into a SIRS category based upon available data (i.e., if a lab value was missing, it was considered to be negative for that parameter and, if present, considered positive if the parameter met positive criteria). Samples for the non-SIRS category were only selected from days that had all available data and were negative for each parameter. Once sorted into the 4 SIRS categories, 115 subjects were randomly selected, based upon sample availability and maintaining a balance between the etiologies of their acute liver failure (acetaminophen toxicity, viral hepatitis or other etiologies). Currently, 46% of all ALF cases (in both the USALFSG and the US) result from APAP toxicity, either suicidal overdoses or unintentional excessive dosing [15]. To ensure a balanced distribution of etiologies, an equal number of samples were requested for each etiology.

In addition, 10 subjects from each of the CLDD and NSTLD repositories were randomly selected as negative controls. These subjects were ambulatory patients with CLD who had provided a screening blood sample during a routine clinic visit without symptoms. Limited bio-data were available for this group. These subjects were considered to be non-SIRS and had no clinical or laboratory evidence of bacterial infection.

### Immunoassay testing

The ADVIA Centaur BRAHMS PCT assay [17] is a sandwich immunoassay utilizing a monoclonal mouse antibody to fluorescein covalently linked to paramagnetic particles, a monoclonal mouse antibody to procalcitonin that is labeled with acridinium ester and a second monoclonal mouse antibody to procalcitonin labeled with fluorescein.

All testing was performed at Siemens Healthcare Diagnostics in Tarrytown, NY on a single ADVIA Centaur. Siemens Healthcare Diagnostics, Tarrytown, NY, provided all reagents required for testing.

### Statistical Analyses

Descriptive statistics (median and range) are provided for all analyzed samples by group. A Kruskal-Wallis test with post hoc testing (Dunn Method) was used to determine the utility of PCT to detect infection, to examine possible measurement differences in PCT between patients with CLD and ALF, and to determine the existence of a dose affect between PCT values and severity of infection in ALF patients [18]. A Chi-square test was used as an alternative method for analyzing patient groups and infection status. PCT values were categorized into infection and non-infection groups using ROC analyses [19]. A 2-way analysis of variance was performed to examine the difference in PCT by type of culture (blood vs. all other culture types), severity categories (severe sepsis and septic shock), and the interaction of these two factors.

The median PCT values for the CLD, non-SIRS, and SIRS groups were compared to the reference range of the PCT assay. An expected reference value of <0.1 ng/mL had been previously calculated from PCT values from a population of 456 normal subjects. The 95% confidence interval for the mean concentration was 0.023 to 0.028 ng/mL [17]. SPSS version 20.0 (SPSS, Chicago, IL) and SAS version 9.2 (Cary, NC) were used for statistical analyses.

## Results

### Study group characteristics based upon disease severity

Available demographic and laboratory data for the 115 study subjects and control groups are shown in Tables 1 and 2. No discernible differences were apparent between the groups studied, except that the CLD group was somewhat older and the overall ALF group contained more women. Laboratory and certain clinical values differed significantly across the various study groups generally reflecting severity of illness: WBC, platelets, INR, AST, ALT, creatinine, lactate, pO_2_, O_2_ saturation, pCO_2_, pulse, respiration, and MAP.

**Table 1:** Demographic Data for Patients Based Upon Severity of Illness Classifications.

Mean (± SD) or	CLD	non-SIRS	SIRS	Severe Sepsis	Septic Shock	
Number (%)	n = 20	n = 30	n = 29	n = 40	n = 16	p-value
Age (years)	51 (29–71)	49 (19–86)	37 (18–66)	41 (17–73)	41 (19–71)	0.044
Gender						
Female	10 (50)	15 (50)	21 (72.4)	33 (82.5)	12 (75)	0.019
Ethnicity*						
Hispanic		5 (16.7)	5 (17.2)	5 (12.5)	1 (6.7)	
Race**						0.672
White	14 (77.8)	21 (66.7)	23 (79.3)	33 (82.5)	14 (87.5)	
African American	1 (5.6)	5 (16.7)	5 (17.2)	4 (10.0)	1 (6.2)	
Asian	2 (11.1)	2 (6.7)	1 (3.4)	2 (5.0)	1 (6.2)	
Hawaiian		1 (3)				
Other	1 (5.6)	1 (3)		1 (2.5)		
Diagnosis						
APAP		10 (33.3)	10 (34.5)	16 (40.0)	5 (31.2)	
Viral Hepatitis	10 (50)	10 (33.3)	10 (34.5)	7 (17.5)	1 (6.2)	
Other	10 (50)	10 (33.3)	9 (31.0)	17 (42.5)	10 (62.5)	
Outcome (TFS)						
APAP		6 (20)	5 (17.2)	12 (30.0)	1 (6.2)	
Viral Hepatitis		4 (13.3)	2 (6.9)	2 (7.4)		
Other		1 (3.3)	4 (13.8)	5 (12.5)	1 (6.2)	
Coma Grade						
Not Reported / 0	20 (100)		4 (13.8)	3 (7.5)		
1		11 (36.7)	1 (3.4)	4 (10.0)		
2		11 (36.7)	8 (27.6)	1 (2.5)		
3		4 (13.3)	5 (17.2)	11 (27.5)	1 (6.2)	
4		4 (13.3)	11 (37.9)	21 (52.5)	15 (93.8)	
Culture Types						
Blood				6	5	
Tracheal				12	4	
Urine				5	3	
Wound				3	1	
Catheter				1		
Multiple w Blood				10	2	
Multiple w/o Blood				3	1	
Antibiotics Used		19 (63.3)	23 (79.3)	33 (82.5)	13 (81.2)	0.294
Prophylaxis		15 (50)	17 (58.6)	19 (47.5)	10 (62.5)	0.673
Therapy		7 (23)	12 (41.4)	22 (55.0)	7 (43.8)	0.069
Age of Sample (years)	9 (1–15)	7 (2–13)	7 (2–13)	6 (2–13)	6.5 (2–11)	0.872
After Hospital Admission (days)		2 (0–11)	3 (0–51)	2 (0–22)	3 (0–16)	0.557

*Ethnicity: O were reported in the CLD group; 15 of 16 were reported in the Septic Shock group

**Race: 18 of 20 reported in the CLD group / p-value reported for white vs. all other races.

**Table 2:** Biodata and Laboratory values for Patients Based Upon Severity of Illness Classifications.

	CLD	non-SIRS	SIRS	Severe Sepsis	Septic Shock	
Median	n = 20	n = 30	n = 29	n = 40	n = 16	p-value
Pulse [bpm]		80	96	108	112	<0.001
Respiration [breaths/min]		18	23	22	22	0.005
MAP [mmHg]		90.0	81.5	89.5	57.5	0.472*
Min Temp [°C]		36.6	35.9	36.5	36.0	0.200
Max Temp [°C]		36.8	36.7	37.4	34.3	0.101
Laboratory values						
PCT [ng/mL]	0.104	1.57	2.29	2.51	5.89	<0.001
AST [IU/L]		787	360	292	3069	<0.001
ALT [IU/L]		1894	775	750	1520	0.030
WBC [x 10^3^ /uL]		8.0	14.0	14.0	17.00	<0.001
Platelets[x 10^3^ /uL]		143	88	115	61	0.002
Prothrombin time [secs]		27.5	25.0	21.0	25.0	0.124
INR [ratio]		3.0	2.0	2.0	3.0	0.030
Bilirubin [mg/dL]		15.0	10.0	10.0	12.5	0.902
Glucose [mg/dL]		122	103	110	145	0.997
Creatinine [mg/dL]		1.00	2.00	1.00	3.00	0.003
Lactate [mg/dL]		3.00	6.00	8.00	14.00	0.024
pO2 [mmHg]		117	86	107	93	0.025
pCO2 [mmHg]		34	27	27	25	<0.001
O2_saturation [%]		98	96	98	95	0.001
FiO2 [%]		28	40	40	50	0.056
pO2/FiO2 [ratio]		334	306.7	306.7	172.5	0.195

*Septic Shock was not included as MAP is defined by the category.

Within the group of 115 ALF patients examined, all 56 with severe sepsis or septic shock had demonstrated positive bacterial cultures (Table 1), and the CLD, non-SIRS, and SIRS groups had no reported positive cultures on or prior to the study day. Blood stream infections (with or without other positive cultures) were present in 23 of the 56, with the majority of the remaining infections being tracheal, urinary tract or multiple without blood stream infection (n = 16, 8 and 4 respectively).

**Fig 1:**
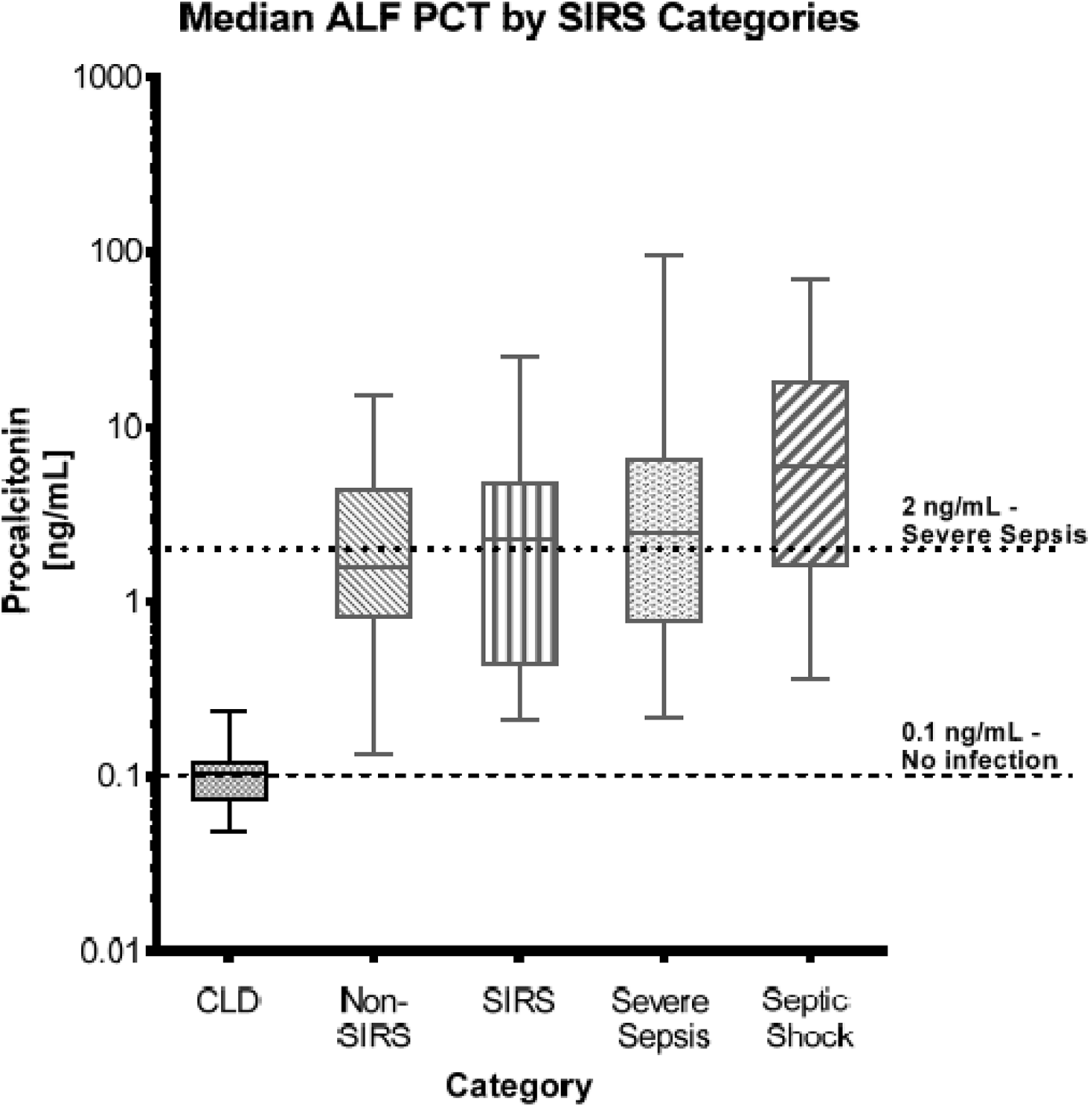
Median PCT Values by Category. This graph represents the median PCT values for the five patient severity groups. The lower dashed horizontal line represents the 0.1 ng/mL PCT cut-off value indicative of no infection. The upper dotted horizontal line represents the 2.0 ng/mL PCT cut-off value indicative of severe sepsis.

Fig 1 and Table 2 present the median results for the PCT assay across all categories. There was a significant difference between the CLD, non-SIRS and SIRS groups with a Chi-square = 43.68 (df = 2, p-value <0.001) as well as between the CLD group and either the non-SIRS or the SIRS groups (both p-values <0.001). However, there was no significant difference between the non-SIRS and SIRS groups (p-value >0.05). When PCT results for CLD, non-SIRS, and SIRS groups combined were compared to the documented reference value of the PCT assay (<0.1 ng/mL—(17)), the median PCT value (0.97 ng/mL) was significantly higher than the reference value (p <0.001). Results for the CLD group (median = 0.104 ng/mL) considered separately did not differ from the published reference range (p = 0.985), while results from each of the other two groups (non-SIRS–median = 1.57 ng/mL and SIRS–median = 2.29 ng/mL) were significantly higher than the reference and the CLD median value (p <0.001 for both).

When PCT concentrations were compared across all categories, there was a significant difference between the CLD group and the four ALF categories (Chi-square = 53.98, p-value <0.001). Post hoc pairwise testing indicated that the median PCT of the CLD group was significantly different from each of the ALF categories (p<0.001). However, there were no significant differences observed (p = 0.169) between the four ALF categories themselves (non-SIRS, SIRS, severe sepsis and septic shock). When we compared the PCT values between ALF subjects without a documented infection (non-SIRS and SIRS) to those with a documented infection (severe sepsis and septic shock), there was no difference between the 2 groups (p = 0.082).

Subjects in severe sepsis and septic shock categories were examined based upon types of cultures: blood cultures vs. all other culture types. There were no significant differences observed for any of the 3 interactions: type of culture (p = 0.224), severity categories (p = 0.309), or the interaction between these two factors (p = 0.241). There was also no difference observed between the median PCT levels for subjects with positive blood cultures and those with positive cultures from other sources.

### Study group characteristics based upon PCT cut-off value

**Fig 2:**
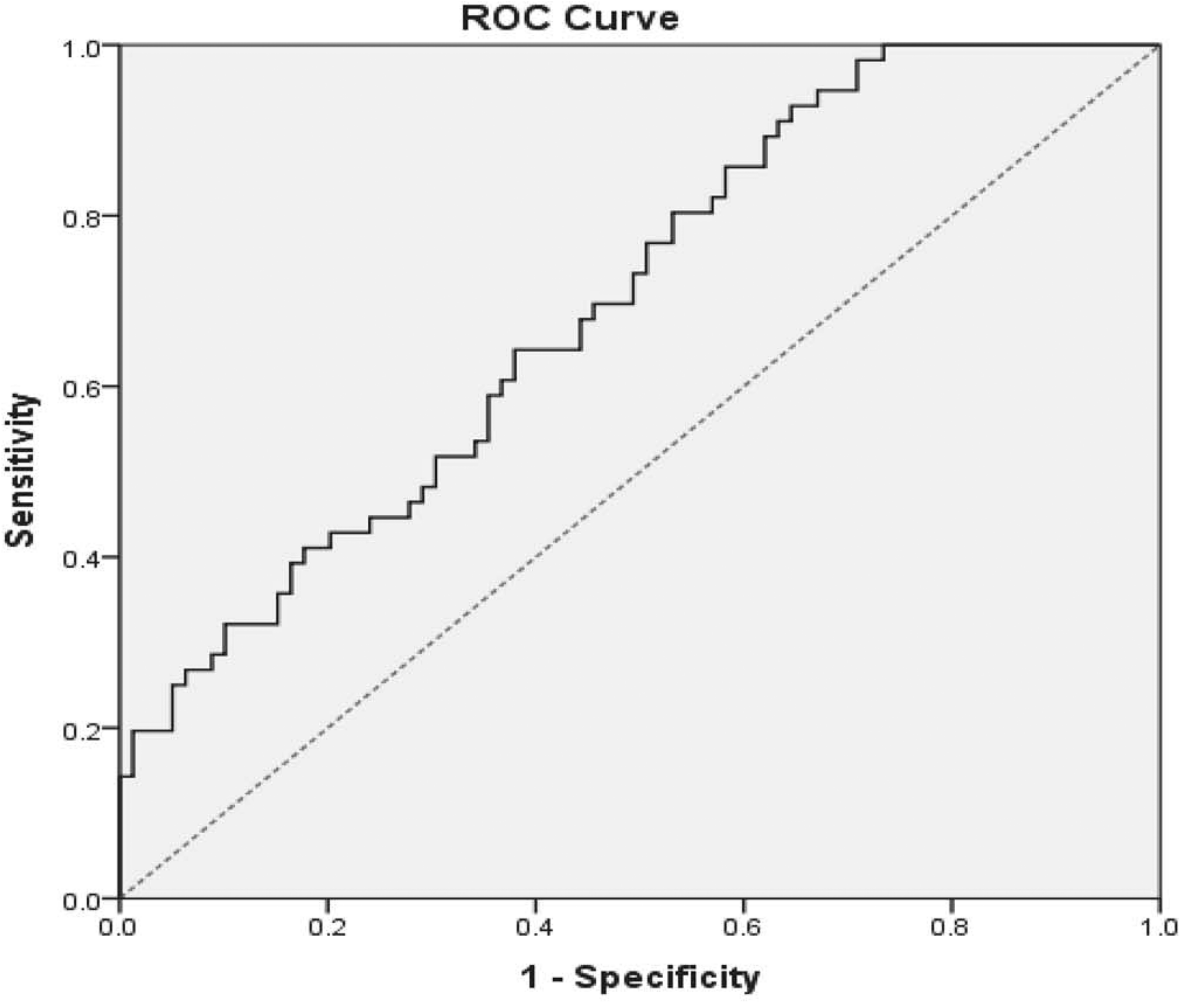
Receiver Operator Curve for the Detection of Infection in ALF and ALI patients Using PCT. The ROC analysis resulted in an AUC of 0.697 with a sensitivity of 64.3% and a specificity of 62.0% for the use of PCT in the detection of infection this population of ALF patients.

In previous studies, values of PCT above 2.0 ng/mL were considered to be associated with bacterial infection. While our non-SIRS and SIRS categories lacked evidence of infection, both demonstrated median PCT values near or slightly above the 2.0 ng/mL cut-off, 1.57 and 2.29 respectively. Given the uncertainty concerning what a reasonable cut-off value for infection should be for this population, we used a receiver operator characteristic (ROC) analysis, in which the optimal area under the curve (AUC) was equal to 0.697 (95% CI: 0.609–0.784), with a cut-off value of 1.62 ng/mL, somewhat below the 2.0 ng/mL level (Fig 2). The cut-off value determined from this set of data was made using a combination of criteria: 1) the point on the curve that was at the greatest distance (0.186) from the 0.5 reference line at a 45° angle to the line, 2) accuracy, 3) sensitivity (0.643), 4) specificity (0.620), and 5) likelihood ratio (1.693). We used this 1.62 ng/mL cut-off for subsequent analyses.

When additional biochemical data ALF between subjects with a PCT that was <1.62 ng/mL and from those with a PCT that was ≥1.62 ng/mL were compared, platelet, creatinine, AST, ALT, and bilirubin showed significant differences between the theoretically ‘infected’ (above the cut-off) and ‘non-infected’ (below the cut-off) groups (Table 3). Of interest, 85.4% of all APAP subjects had values above the 1.62 ng/mL level while only 21 of 41 (51.2%) of the APAP subjects had a documented bacterial infection. In the non-APAP subjects, 43.2% had values above 1.62 ng/mL with documented bacterial infection in 35 of 74 (47.3%) of the subjects.

**Table 3:** Demographic and Biodata Results Based Upon the Calculated Infection Cut-off Value of 1.62 ng/mL.

Median or Number (%)	PCTs < 1.62 ng/mL n = 48	PCT ≥ 1.62 ng/mL n = 67	p-value
ALF Etiology			
APAP	6 (12.5)	35 (52.2)	
Viral hepatitis	17 (34.7)	11 (16.4)	
Other	25 (51.0)	21 (31.3)	
Outcome (TFS)			
APAP	3 (6.2)	21 (31.3)	
Viral Hepatitis	5 (10.4)	3 (4.4)	
Other	5 (10.4)	6 (9.0)	
Procalcitonin [ng/mL]	0.6904	5.325	<0.001
WBC [x 10^3^ /uL]	13.0	13.0	0.740
Platelets [x 10^3^ /uL]	135	92	0.019
INR [ratio]	2.0	2.0	0.786
Glucose [mg/dL]	115.5	120.0	0.787
Creatinine [mg/dL]	1.0	2.0	<0.001
AST [IU/L]	337	913	0.06
ALT [IU/L]	684	1759	0.07
Lactate [mg/dL]	3.5	6.0	0.230
Bilirubin [mg/dL]	19.0	7.0	<0.001
pO2 [mmHg]	92.5	103.0	0.707
pCO2 [mmHg]	29.0	27.0	0.108
Pulse [bpm]	84	68	0.084
Respiration [breaths/min]	17	13	0.094
Min Temp [°C]	36.0	37.0	0.298
Max Temp [°C]	37.0	37.0	0.298
Coma Grade			
0 / Not reported	3	4	
1	7	9	
2	12	8	
3	9	12	
4	18	33	

Samples were resorted based upon the cut-off value of 1.62 ng/mL and demographic and biodata were re-examined.

**Fig 3:**
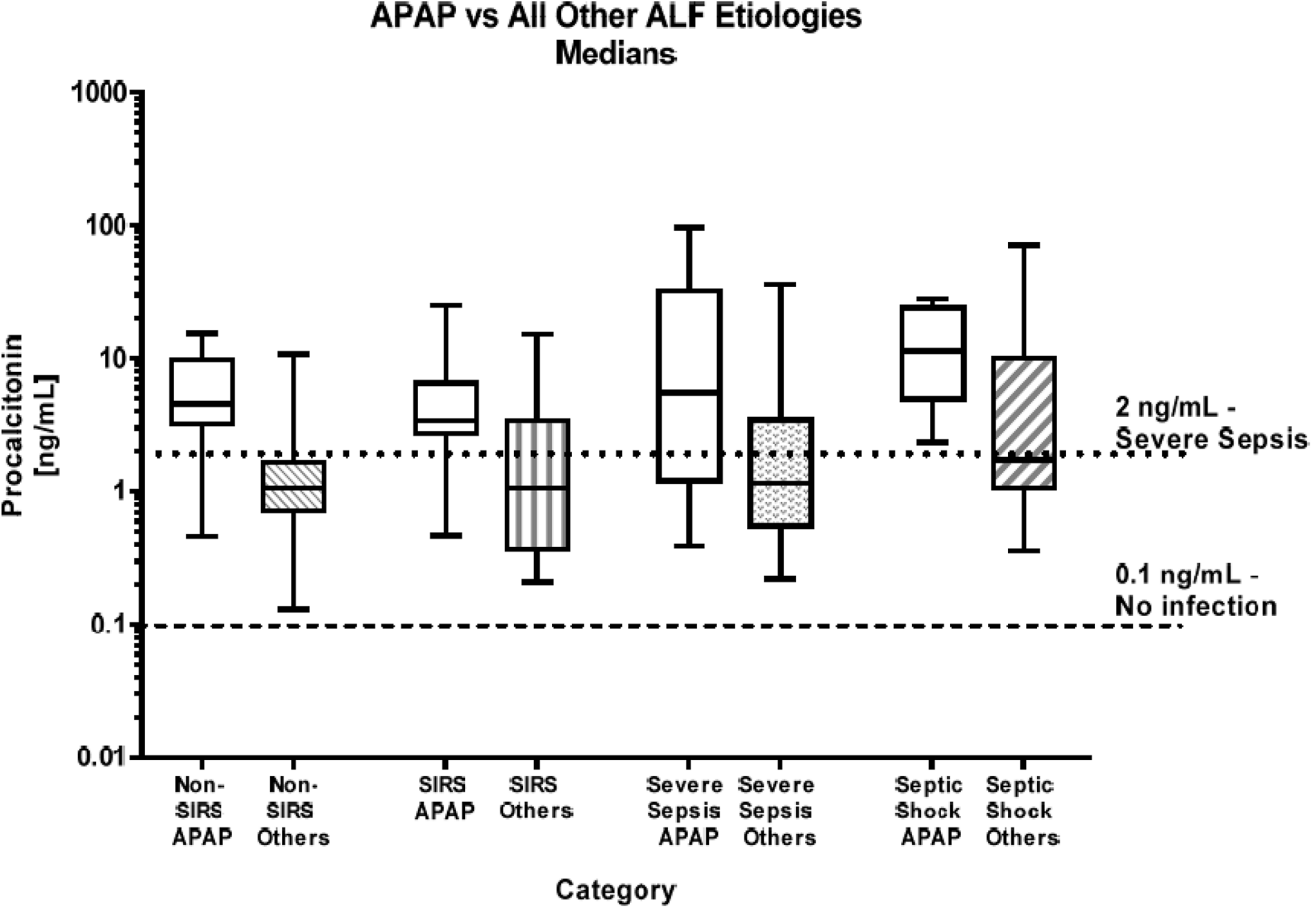
Median APAP vs All Other Etiologies PCT values by Category. This graph represents the median PCT values for the four ALF patient severity groups sorted by etiologies: APAP and All Others etiologies (combined viral and other) with the lower dashed line indicative of no infection (0.1 ng/mL) and the upper dotted line indicative of severe sepsis (2.0 ng/mL).

When PCT results were examined based solely upon etiology, median values for subjects with APAP toxicity were typically much higher, between 3.0 and 6.6 times higher than the results observed for the same severity category for subjects with non-APAP etiologies (Fig 3).

## Discussion

In this study of ALF patients selected for increasing severity of SIRS features and for the presence of bacterial infection, PCT measurement disclosed markedly elevated values but did not provide any direct correlation to presence or absence of infection. Determining the presence with certainty of systemic infection in those with advance SIRS features is problematic in a retrospective study; we assumed that the presence of advanced SIRS features plus positive cultures was the best surrogate for systemic infection and this may not be the case in every instance. However, virtually all ALF subjects displayed PCT values well above both the 0.1 ng/mL reference value and the median value observed in the CLD controls, regardless of any evidence for infection, particularly high values being observed in a large group that demonstrated no evidence for infection. The differences in PCT levels between severity categories among ALF patients were not significant due apparently to elevated levels in the absence of infection. Thus, both the non-SIRS and SIRS categories that would be expected to show low or normal PCT levels demonstrated PCT levels approximating those that have been observed previously in other studies during active bacterial infection in a non-ALF setting.

Using a cut-off value determined by the ROC analysis, the presumed uninfected categories (non-SIRS and SIRS) included 31 patients with elevated PCTs (≥1.62 ng/mL). Of these subjects, 13 demonstrated signs of infection (bacterial or fungal, n = 5 each) 1 or more days after the PCT sample was collected or other evidence of infection upon closer examination of the each subject’s case report form (CRF), including pancreatitis, tooth abscess or ventilator acquired pneumonia with infiltrate on x-ray. Subjects with fungal infections were classified as culture negative since values of PCT in yeast and fungal infections have been observed to be lower than those seen with most bacterial infections [20].

The median PCT values in the severe sepsis and septic shock categories (3.46 ng/mL and 589 ng/mL, respectively), were both well above the 1.62 ng/mL calculated cut-off and 2.0 ng/mL literature cut-off for severe sepsis [10], but these were not significantly different from the median PCT values in non-SIRS and SIRS patients without documented infection. Of interest, 17 of 40 severe sepsis subjects and 3 of 16 septic shock subjects had PCT values below the 1.62 ng/mL cut-off. Prophylactic antibiotics might provide a partial explanation for lower PCT values during a documented infection but, overall, the decreased results remain unexplained. Full bacterial culture reports were not collected as part of the USALFSG study nor did the study case report forms capture information related to quantity of bacterial growth or antibiotic sensitivities that might have helped determine some the differences among the various severity groups.

Upon examining results according to etiology, subjects with APAP toxicity demonstrated much higher PCT levels than any other etiologies. It seems likely that the elevated PCT values in these subjects are not indicative of infection. Rather, APAP toxicity unleashes an inflammatory response, with macrophage activation, release of cytokines and pro-inflammatory regulators including TNF-alpha that may secondarily contribute to liver injury and the appearance of a septic milieu [21, 22]. While the exact origin and pathogenic pathway of PCT stimulation is still unknown, it has been suggested that PCT is produced by neuroendocrine cells in the liver while other data suggest that it is released by macrophages [23]. It is also possible that increases in PCT may be initiated by TNF-alpha and IL-1b [24, 25]. It appears that in the acute liver failure setting PCT is increased due in large part to the massive inflammatory response to liver injury, particularly in the case of acetaminophen toxicity. While we did see higher PCT levels in patients with bacterial infections, if PCT is to be useful in this population, a better understanding of the mechanism of PCT production will need to be ascertained.

Prior studies examining PCT values in the acute liver failure setting have been limited. Studies in patients with chronic liver disease (cirrhosis) [26–29], liver metastasis of non-liver primary solid tumors [23], and other acute and chronic liver diseases have been performed [30]. While baseline PCT values increased as the severity of the underlying liver disease increased, an elevated PCT was indicative of bacterial infection. A previous study of PCT use in ALF patients due to APAP toxicity also showed increased PCT values in subjects without evidence of bacterial infection [14], similar to our findings.

We considered whether the use of older retrospective samples from the ALFSG repository might affect results, since long-term sample storage may decrease observed PCT levels. Schuetz, et al. studied two sets of samples that were stored at -80°C for 4.8 to 5.5 years and 3.3 to 4.6 years [31]. The PCTs for each set decreased by 11.4% and 13.5% respectively. Samples used in this study had been stored an average of approximately 6.75 years at -80°C. While it is assumed that the length of time in storage might have decreased the PCT values obtained, the exact amount of decrease is unknown and an estimate of the extent of the effect cannot be calculated. However, since each sample group had similarly aged samples, this would appear to negate a specific effect on the overall study results (Table 1).

## Conclusion

Procalcitonin detects bacterial infection and sepsis in the general population, but appears to be a more general marker of moderate to severe inflammation. As such, it has limited value as a single marker to discriminate between the presence or absence of bacterial infection in the setting of acute liver failure but may be of value in combination with other markers. PCT elevations appeared to be roughly proportional to the degree of hepatic necrosis. A prospective study with a larger sample size employing prospective determination of infection and standardized antibiotic usage might provide additional information regarding the relationship between PCT and severe liver injury, particularly APAP toxicity.

## Supporting Information

S1 Data Study Clinical and Laboratory Data. (XLSX)

## Abbreviations

ALF: acute liver failure
ALT: alanine aminotransferase
APAP: acetaminophen
AST: aspartate aminotransferase
AUC: area under the receiver operator characteristic curve
CLD: chronic liver disease
CRF: case report form
HBV: hepatitis B virus
HE: hepatic encephalopathy
INR: International Normalized Ratio of prothrombin time
LLD: lower limit of detection
LT: liver transplantation
MAP: mean arterial pressure
PCT: procalcitonin
PT: prothrombin time
ROC: receiver operator characteristic curve
SIRS: systemic inflammatory response syndrome
TFS: transplant-free survival
WBC: white blood cell count

## References

[1] Rolando N, Wade J, Davalos M, Wendon J, Philpott-Howard J, Williams R. The systemic inflammatory response syndrome in acute liver failure. Hepatology, 2000; 32(4): 734–739. 11003617 10.1053/jhep.2000.17687

[2] Rolando N, Wade J, Davalos M, Wendon J, Philpott-Howard J, Williams R. Timing and aetiology of bacterial infections in a liver intensive care unit. J Hosp Infect, 2003; 53(2): 144–146. 12586576 10.1053/jhin.2002.1363

[3] Stravitz RT. Critical management decisions in patients with acute liver failure. Chest, 2008; 134: 1092–1102. 10.1378/chest.08-1071 18988787

[4] Vaquero J, Polson J, Chung C, Helenowski I, Schiodt FV, Reisch J, et al Infection and the progression of hepatic encephalopathy in acute liver failure. Gastroenterology, 2003; 125(3): 755–764. 12949721 10.1016/s0016-5085(03)01051-5

[5] Becker KL, Nylen ES, White JC, Muller B, Snider RH, Jr. Procalcitonin and the calcitonin gene family of peptides in inflammation, infection, and sepsis: A journey from calcitonin back to its precursors. J Clin Endocrinol Metab, 2004; 89(4): 1512–1525. 15070906 10.1210/jc.2002-021444

[6] Bracq S, Machairas M, Clement B, Pidoux E, Andreoletti M, Mokhtar MS, et al Calcitonin gene expression in normal human liver. FEBS Letters, 1993; 331(1,2): 15–18. 8405394 10.1016/0014-5793(93)80288-6

[7] Carrol ED, Thomson APJ, Hart CA. Procalcitonin as a marker of sepsis. Int J Antimicrob Agents, 2002; 20: 1–9. 12127705 10.1016/s0924-8579(02)00047-x

[8] Muller B, Becker KL. (2001). Procalcitonin: How a hormone became a marker and mediator of sepsis. Swiss Medi Wkly, 2001; 131(41–42): 595–602. 10.4414/smw.2001.09751 11820070

[9] Brunkhorst FM, Wegscheider K, Forycki ZF, Brunkhorst R. Procalcitonin for early diagnosis and differentiation of SIRS, sepsis, severe sepsis, and septic shock. Intensive Care Med, 2000; 26: S148–S152. 10.1007/BF02900728 18470710

[10] Harbarth S, Holeckova K, Froidevaux C, Pittet D, Ricou B, Grau GE, et al Diagnostic value of procalcitonin, interleukin-6, and interleukin-8 in critically ill patients admitted with suspected sepsis. American Journal of Respiratory Critical Care Medicine, 2001; 164: 396–402. 11500339 10.1164/ajrccm.164.3.2009052

[11] Brunkhorst FM, Eberhard OK, Brunkhorst R. Discrimination of infectious and noninfectious causes of early acute respiratory distress syndrome by procalcitonin. Crit Care Med, 1999; 27(10): 2172–2176. 10548201 10.1097/00003246-199910000-00016

[12] Luyt C, Guerin V, Combes A, Trouillet J, Ayed SB, Bernard M, et al Procalcitonin kinetics as a prognostic marker of ventilator-associated pneumonia. Am J Respir Crit Care Med, 2005; 171: 48–53. 15447947 10.1164/rccm.200406-746OC

[13] Meisner M, Tschaikowsky K, Hutzler A, Schick C, Schuttler J. Postoperative plasma concentrations of procalcitonin after different types of surgery. Intensive Care Med, 1998; 24: 680–684. 9722037 10.1007/s001340050644

[14] Jackson N, Batouche S, Sherwood R, Wendon J. Serial plasma procalcitonin levels in patients requiring admission to liver ITU with paracetamol induced acute liver failure. Abstract Brit J Anaesth, 2000; 84 (5): 92P.

[15] Lee WM, Squires RH Jr, Nyberg SL, Doo E, Hoofnagle JH. Acute liver failure: Summary of a workshop. Hepatology, 2008; 47: 1401–1415. 10.1002/hep.22177 18318440 PMC3381946

[16] Levy MM, Fink MP, Marshall JC, Abraham E, Angus D, Cook D, et al 2001 SCCM/ESICM/ACCP/ATS/SIS international sepsis definitions conference. Crit Care Med, 2003; 31(4): 1250–1256. 12682500 10.1097/01.CCM.0000050454.01978.3B

[17] Siemens Healthcare Diagnostics, Ltd. *Advia centaur and advia centaur XP systems—procalcitonin (PCT)*. Frimley, Camberley, UK; 2010. Rev B.

[18] Elliot AC, Hynan LS. A SAS^®^ macro implementation of a multiple comparison post hoc test for a Kruskal-Wallis analysis. Comput Methods Programs Biomed, 2011; 102(1): 75–80. 10.1016/j.cmpb.2010.11.002 21146248

[19] Riffenburgh RH. Statistics in Medicine. Second Edition New York, NY, Elsevier, 2006.

[20] Nakamura A, Wada S, Takeda T, Nobori T. Efficacy of procalcitonin in the early diagnosis of bacterial infections in a critical care unit. Shock, 2009; 31(6): 586–591. 10.1097/SHK.0b013e31819716fa 19060784

[21] Dragomir A-C, Laskin JD, Laskin DL. Macrophage activation by factors released from acetaminophen-injured hepatocytes: Potential role of HMGB-1. Toxicol Appl Pharmacol, 2011; 253: 170–177. 10.1016/j.taap.2011.04.003 21513726 PMC3507385

[22] Jaeschke H, McGill MR, Ramachandran A. Oxidant stress, mitochondria, and cell death mechanisms in drug-induced liver injury: Lessons learned from acetaminophen hepatotoxicity. Drug Metab Rev, 2012; 44(1): 88–106. 10.3109/03602532.2011.602688 22229890 PMC5319847

[23] Matzaraki V, Alexandraki KI, Venetsanou K, Piperi C, Myrianthefs P, Malamos N, et al Evaluation of serum procalcitonin and interleukin-6 levels as markers of liver metastasis. Clin Biochem, 2007; 40: 336–342. 17306245 10.1016/j.clinbiochem.2006.10.027

[24] Whang KT, Vath SD, Nylen ES, Muller B, Li Q, Tamarkin L, et al Procalcitonin and proinflammatory cytokine interactions in sepsis. Shock, 1999; 12(4): 268–273. 10509628 10.1097/00024382-199910000-00004

[25] Domenech VS, Nylen ES, White JC, Snider RH Jr, Becker KL, Landmann R, et al Calcitonin gene-related peptide expression in sepsis: Postulation of microbial infection-specific response elements within the calcitonin I gene promoter. J Investig Med, 2001; 49(6): 514–521. 11730087 10.2310/6650.2001.33628

[26] Bota DP, Van Nuffelen M, Zakariah AN, Vincent JL. Serum levels of C-reactive protein and procalcitonin in critically ill patients with cirrhosis of the liver. J Lab Clin Med, 2005; 146(6): 347–351. 16310518 10.1016/j.lab.2005.08.005

[27] Connert S, Stremmel W, Eising C. Procalcitonin is a valid marker of infection in decompensated cirrhosis. Abstract Z Gastroenterol, 2003; 41(2): 165–170. 10.1055/s-2003-37314 12592597

[28] Spahr L, Morard I, Hadengue A, Vadas L, Pugin J. Procalcitonin is not an accurate marker of spontaneous bacterial peritonitis in patients with cirrhosis. Hepatogastroenterology, 2001; 48(38): 502–505. 11379342

[29] Viallon A, Zeni F, Pouzet V, Lambert C, Quenet S, Tardy B, et al Serum and ascitic procalcitonin levels in cirrhotic patients with spontaneous bacterial peritonitis: Diagnostic value and relationship to pro-inflammatory cytokines. Intensive Care Med, 2000; 26: 1082–1086. 11030164 10.1007/s001340051321

[30] Elefsiniotis IS, Skounakis M, Vezali E, Pantazis KD, Petrocheilou A, Pirounaki M, et al Clinical significance of serum procalcitonin levels in patients with acute or chronic liver disease. Eur J Gastroenterol Hepatol, 2006; 18(5): 525–530. 16607149 10.1097/00042737-200605000-00012

[31] Schuetz P, Christ-Crain M, Huber AR, Muller B. Long-term stability of procalcitonin in frozen samples and comparison of kryptor and VIDAS automated immunoassays. Clin Biochem, 2010; 43(3): 341–344. 10.1016/j.clinbiochem.2009.08.029 19747473

